# Prediction of the potentially suitable areas of *Paeonia lactiflora* in China based on Maxent and Marxan models

**DOI:** 10.3389/fpls.2024.1516251

**Published:** 2025-01-09

**Authors:** Yongji Wang, Wentao Huo, Kefan Wu, Jiaying Cao, Guanghua Zhao, Fenguo Zhang

**Affiliations:** ^1^ School of Life Science, Shanxi Engineering Research Center of Microbial Application Technologies, Shanxi Normal University, Taiyuan, Shanxi, China; ^2^ School of Life Science, South China Normal University, Guangzhou, China

**Keywords:** *Paeonia lactiflora*, climate change, Maxent, Marxan, prediction of suitable area

## Abstract

*Paeonia lactiflora* Pall. (*P. lactiflora*) is an important medicinal plant in China with high ornamental value. Predicting the potential habitat of *P. lactiflora* is crucial for identifying its geographic distribution characteristics and ensuring its ecological and economic importance. Therefore, we aimed to predict the potential geographic distribution of *P. lactiflora* in China under future climate change scenarios. To this end, we used an optimized Maxent model and ArcGIS software to analyze the influence of 12 environmental variables on *P. lactiflora* potential distribution in China based on 291 effective distribution records. The key factors limiting the potential geographic distribution of *P. lactiflora* were evaluated by combining the contribution rates of the environmental variables with the significance of their replacement. The jackknife method was employed to assess the importance of these factors. Response curves were used to determine the appropriate intervals for the environmental factor variables and to analyze the changes in spatial patterns. The Maxent model exhibited a low degree of overfitting and good prediction accuracy. The main variables influencing *P. lactiflora* distribution were precipitation in the wettest month and hottest quarter, lowest temperature in the coldest month, and highest temperature in the warmest month. Under current climatic conditions, *P. lactiflora* could theoretically grow across and area of 231.1 × 10^4^ km^2^ in China. Under the six future climate change scenarios, the potential geographic distribution area was reduced compared with the current distribution area, and the potentially suitable areas shifted southwestward. The majority of priority conservation sites for *P. lactiflora* are located in northern and northeastern China, which align with the highly favorable areas predicted by the Maxent model. The findings of this investigation can guide the selection of future introductions as well as artificial cultivation and preservation of *P. lactiflora* resources.

## 1 Introduction

Global warming is increasing due to the intensity of human activities (Klein and Anderegg, 2021). The Sixth Assessment Report of the United Nations Intergovernmental Panel on Climate Change states that between 2011 and 2020, the temperature increased by 1.09°C (range of 0.95–1.20°C) compared to that during 1850–1900 (Zhou, 2021). Global warming has had far-reaching effects on plant communities. Studies have suggested that climate change-induced habitat destruction may become the greatest global threat to biodiversity in the coming decades (Li et al., 2019). As global warming accelerates, some species will migrate to higher latitudes or elevations, whereas others may adapt physiologically or phenologically to changes in their habitat (Root et al., 2003; Zhang et al., 2018). Research on the influence of climate change on species’ potential habitats has prompted efforts toward safeguarding rare plant resources and facilitating the introduction and growth of economically important plants. Consequently, this area of research has gained momentum, focusing on understanding the ramifications of global change on species distribution (Zhao et al., 2021). Furthermore, because the climate varies with latitude in terms of temperature and precipitation, it has a substantial impact on species distribution, in conjunction with other environmental factors, including terrain, soil type, and biotic interactions (Puchałka et al., 2023). Understanding how species have responded to climate change in the past and how they will respond in the future can assist scientists in managing germplasm resources and comprehending the historical factors that have led to the emergence of new species and changes in their geographic ranges (Zhao et al., 2021).

Ecological niche modeling quantifies the correlation between species occurrence and various environmental variables to characterize the ecological niche or habitat suitability of a species (Guisan and Zimmermann, 2000). It is an effective tool for explaining changes in species distribution due to environmental variables such as climate (Zimmemann et al., 2011). Appropriate plant distribution areas can be estimated by examining the features of plant ecological niches and associated environmental factors in relation to the known geographic distribution of plants (Zhao et al., 2021). Various species distribution models, including CLIMEX, Domain, Genetic Algorithm for Rule-Set Production, and Maximum Entropy (Maxent), have been utilized to assess the ecological requirements, responses, and distribution areas of species (Guisan and Thuiller, 2004; Phillips and Dudík, 2008). In particular, the Maxent model has been used extensively both regionally and internationally in many fields owing to its intuitive modeling, high prediction accuracy, ease of operation, and strong explanatory power (Wang et al., 2023). This model determines the primary factors and adaptation ranges that affect species growth by combining various environmental variables to predict potential distributions. It is frequently used to study ecological characteristics and forecast the possible distribution of species (Wang et al., 2023a). Puchałka et al. (2023) Using Maxent successfully estimated climate niche shifts and threat levels under various climate change scenarios for two *Vaccinium* species.

*Paeonia lactiflora* Pall., commonly known as the Chinese peony or garden peony, is an herbaceous plant belonging to the Paeoniaceae family. It has been referred to as the “flower of fortune” since ancient times and has substantial economic, ornamental, and medicinal value. As a traditional Chinese flower, *P. lactiflora* boasts large flowers, a beautiful appearance, and a rich fragrance (Zhang et al., 2021; Yuan et al., 2023). In addition, *P. lactiflora* is used for the treatment of inflammatory and cardiovascular diseases and as a neuroprotective agent (Li et al., 2021). Its root is widely used in traditional Chinese medicine, where it is known as “white peony,” due to its high content of terpenoids, flavonoids, polysaccharides, polyphenols, and other bioactive compounds, which have effects such as analgesia, spasmolysis, removal of blood stasis, and promotion of menstruation (Yan et al., 2021). Owing to its aesthetic, therapeutic, and commercial value, *P. lactiflora* has been extensively cultivated worldwide (Xue et al., 2018; Zhao et al., 2019).

Research on this economically important medicinal plant has primarily focused on its chemical composition and pharmacological effects. To our knowledge, this study is the first to integrate the Maxent model with the Marxan framework to predict the distribution of suitable habitats for peonies, identify the key environmental factors influencing this distribution, and provide recommendations for conservation areas. We utilized the optimized Maxent model to enhance the prediction of current and future suitable zones for *P. lactiflora* by incorporating additional environmental factors and minimizing overfitting. In addition, the Marxan model was employed to delineate protected areas to address the endangerment of wild *Paeonia* species. Thus, the goals of this study were to: (1) predict the distribution patterns of potentially suitable areas for *P. lactiflora* in China under current climatic conditions and classify them into different suitability grades; (2) determine the primary environmental variables affecting *P. lactiflora* geographical distribution; (3) forecast and compare the potentially suitable areas for *P. lactiflora* and the changing trends under future climatic conditions; and (4) outline key conservation areas and areas conducive to *P. lactiflora* growth. This analysis can serve as a theoretical foundation for the introduction, propagation, preservation, and sensible use of *P. lactiflora*.

## 2 Materials and methods

### 2.1 Collection of occurrence data

**Figure 1:**
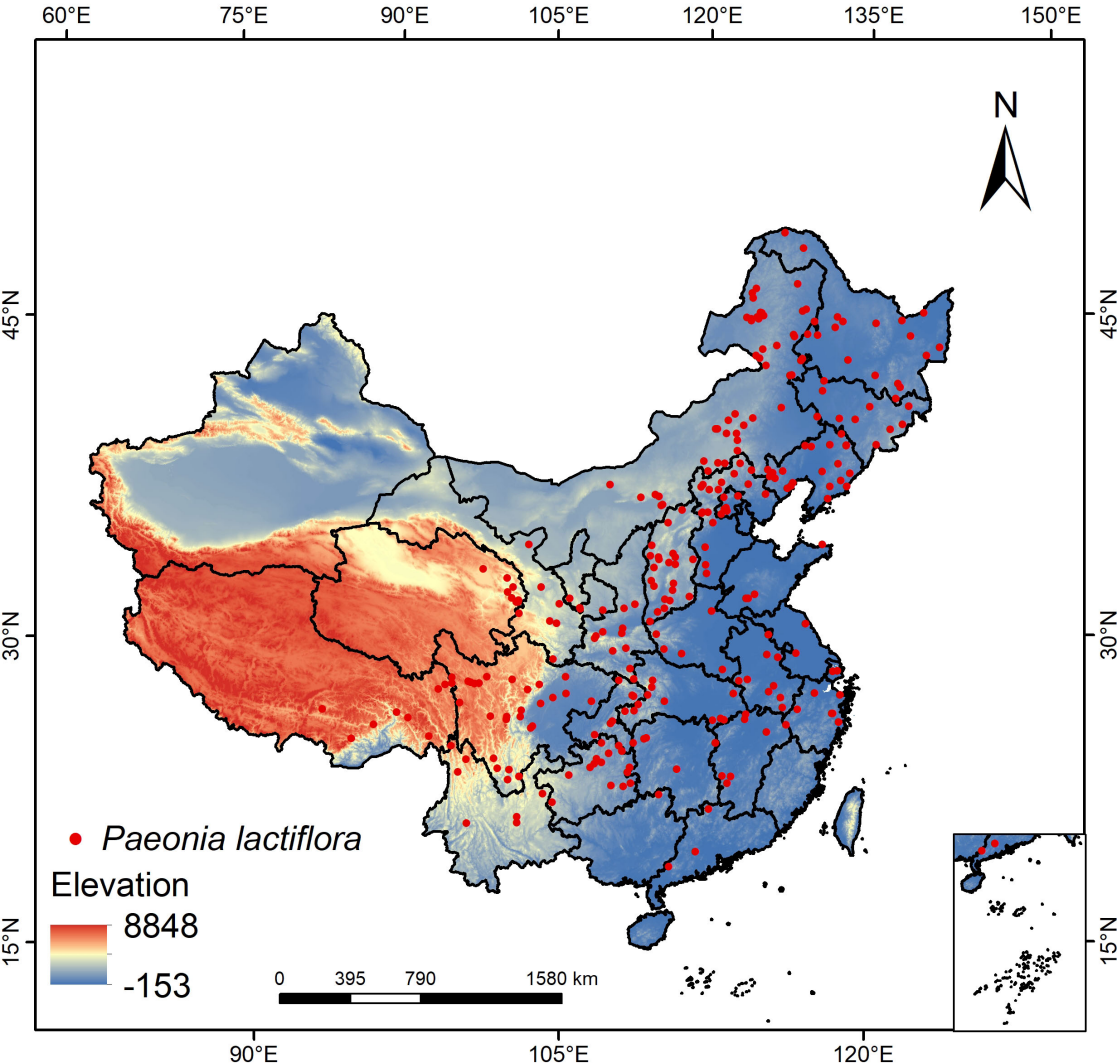
Distribution of occurrence points of *P. lactiflora*.

To gather comprehensive data on the occurrence of *P. lactiflora* across its range, we conducted an exhaustive search of online databases for relevant literature, specifically utilizing the Chinese Virtual Herbarium (CVH, http://www.cvh.org.cn/) and the Global Biodiversity Information Facility (GBIF, https://www.gbif.org/) databases. We utilized the Baidu coordinate retrieval system (https://api.map.baidu.com/lbsapi/getpoint/) to ascertain the latitude and longitude of records that lacked precise geographic coordinates. Duplicate coordinate points, excessively dense species distribution data within a region, and distribution data containing incorrect or incomplete coordinate information were defined and excluded as unqualified data. To reduce sampling bias, we used the R software package “spThin” to filter the data (Aiello-Lammens et al., 2015), retaining only one coordinate point in a 5 km × 5 km grid. After removing incorrect and incomplete coordinate data, and applying the aforementioned filtering method. Ultimately, as illustrated in **Figure 1**, 291 confirmed presence data of *P. lactiflora* were obtained in order to build the models.

### 2.2 Environmental factors

Initially, we identified 38 environmental variables that potentially influence the distribution of *P. lactiflora*. These encompassed three topographical variables and nineteen climatic variables from various time periods, all sourced from the WorldClim 2.1 Database (https://www.worldclim.org), with a resolution of 2.5′ (5 km × 5 km). The averages for the following periods were calculated: present (covering 1970–2000), 2050s (2041–2060), and 2090s (2081–2100). Scenarios combining the Shared Socioeconomic Pathway (SSP) and Representative Concentration Pathway (RCP) based on CMIP6 for projecting future climate variables were developed using the China (Beijing) Climate Center Climate System Model 2 Medium Resolution (BCC-CSM2-MR) (Jiang et al., 2020). CMIP6 addresses the limitations of CMIP5, which just takes into account CO_2_ concentrations and radiative forcing targets, by integrating common socioeconomic scenarios and land use. It provides a more rigorous and scientific explanation of potential future climate scenarios (Ostad-Ali-Askari et al., 2020; Shi et al., 2023). In our study, we considered sustainable development (SSP126), intermediate development (SSP245), and conventional development (SSP585). Additional data on 16 soil factors (basic saturation, carbonate or lime content, sulfate content, cation exchange capacity of cohesive soil, cation exchange capacity of soil, clay content, volume percentage of crushed stone, exchangeable sodium salt, conductivity, organic carbon content, pH, soil bulk density, sand content, silt content, classification of exchangeable base, USDA soil texture) were sourced from the World Soil Database (https://www.fao.org/). Provincial national vector image was acquired from the Ministry of Natural Resources of China (http://www.mnr.gov.cn/).

To address the potential issue of overfitting in the model, which may arise from an abundance of environmental variables and subsequently compromise prediction accuracy, we utilized pertinent R code to analyze multicollinearity and calculate Pearson’s correlation coefficients for the 38 environmental variables. Based on the outcomes, we excluded variables with correlation coefficients |r| ≥0.7. Following this, we selected the variance inflation factor (VIF) as a criterion for further model refinement (Dormann et al., 2013; Zhao et al., 2021b). The variance inflation factor (VIF) is also known as the reciprocal of the tolerance metric. A VIF value below 5 indicates no significant multicollinearity among factors; a VIF ranging from 10 to 100 suggests moderate multicollinearity; and a VIF above 100 indicates substantial interfactor multicollinearity (Wang et al., 2023b). The first environmental factors we considered were those with correlations less than 0.7 and VIF values less than 5. Additionally, we enabled jackknife resampling in the environmental parameter settings to evaluate the weight of the environmental factors. We identified the dominant environmental component by summing the percentage contributions of each environmental factor and the significance of the substitution value (Phillips et al., 2006). We loaded all the environmental factors, along with the *P. lactiflora* distribution data, into Maxent. We allocated 25% of the dataset for testing and used the remaining 75% for training. We set up 10 repetitions, selected Bootstrap as the repetition type, and output the distribution values in logistic form. Ultimately, we selected 12 major environmental variables to model the Maxent model (**Table 1**).

**Table 1:** Environmental factors.

Type	Variable code	Environmental factor	Unit
Climatic factor	bio5	Max Temperature in Warmest Month	°C
	bio6	Min Temperature in Coldest Month	°C
	bio13	Precipitation in Wettest Month	mm
	bio14	Precipitation in Driest Month	mm
	bio18	precipitation in warmest quarter	mm
	bio19	precipitation in coldest quarter	mm
Topographic factors	elev	Altitude	m
	slope	Slope variability	%
Soil factor	t_bs	Basic saturation	%
	t_cec_clay	Cation exchange capacity of cohesive soil	%
	t_esp	Exchangeable sodium salt	%
	t_silt	Silt content in topsoil	%

### 2.3 Model optimization and accuracy evaluation

By optimizing the regulation multiplier (RM) and the feature combination (FC) adjusted using the ENMeval data package in R software, the model’s prediction accuracy can be greatly increased and overfitting can be mitigated (Phillips et al., 2017; Guo et al., 2022). Our research began by analyzing the presence-only datasets available within the ENMeval framework, with the objective of determining the optimal feature class transformations and regularization coefficients. ENMeval facilitates the development of environmental niche models (ENMs) using the presence-only approach in Maxent (Phillips et al., 2006). The Maxent framework comprises five features: linear (L), quadratic (Q), fragmented (H), product (P), and threshold (T) characteristics (Zhao et al., 2021a). In this study, the default parameters of the Maxent software were set to RM = 1 and FC = LQHPT. To refine the Maxent model, the RM was adjusted to value ranging from 0.5 to 4, in increments of 0.5, resulting in a total of eight regulated frequency adjustments. Six pairs of one or more attributes were selected simultaneously: L, L and Q, H, L, Q, H and H, L, Q, H and P, and L, Q, H, P and T. A total of 48 parameter combinations were calculated using permutations and combinations. These 48 parameters were then combined and tested to evaluate the model’s complexity. The evaluation was based on the values of delta. AICc and (auc.train-auc.diff.avg). The model’s prediction accuracy increases as these two values decrease (Zhao et al., 2021b).

We employed the receiver operating characteristic (ROC) curve and the area under the curve (AUC) as measures to assess the model’s accuracy (He et al., 2023). The AUC value ranges from 0 to 1, with higher values indicating a higher degree of confidence in the prediction results. This metric is independent of the model’s threshold. It serves primarily as a metric to evaluate the accuracy of the model (Poririo et al., 2014; Wan et al., 2021). An AUC value closer to 1 indicates a higher level of prediction accuracy. Specifically, an AUC between 0.5 and 0.7 suggests a moderate prediction, between 0.8 and 0.9 indicates a good prediction, and between 0.9 and 1.0 signifies an excellent prediction (Dakhil et al., 2019; Ren et al., 2020). The True Skill Statistic (TSS) serves as an indicator of the net prediction success rate, encompassing both presence and absence data, and has been widely applied in various ecological models in recent years. TSS values range from -1 to +1, where +1 denotes perfect agreement, and values of 0 or lower reflect performance that is indistinguishable from random. The closer the TSS value is to 1, higher the level of prediction accuracy (Allouche and Kadmon, 2006).

### 2.4 Changes in the spatial pattern of the suitable distribution area for *P. lactiflora*

Based on the methodology outlined by Zhang’s et al. (2019), the probabilistic outcomes of suitable distribution areas of *P. lactiflora* obtained from the model were reclassified. To establish a binary presence/absence matrix (0, 1) for the potential geographical distribution of *P. lactiflora* under both current and future climate change scenarios, spatial units with a probability of species existence ≥ 0.49 were classified as suitable areas, whereas those with a probability < 0.49 were deemed unsuitable areas. Suitable areas were assigned the value of “1”, whereas the unsuitable areas were assigned the value of “0”. Further analysis was conducted regarding the spatial pattern of the suitable distribution zones for *P. lactiflora* under future climate change scenarios, using the presence/absence matrix. Consequently, four categories of changes in suitable areas were identified: unsuitable area, lost suitable area, reserved suitable area, and newly added suitable area. These categories facilitate an understanding of how past and future climate change will modify the spatial patterns of potentially suitable areas: An unsuitable area is represented by a matrix value of 0 → 0. A newly added suitable area is indicated by a value of 0 → 1. A lost suitable area is indicated by a value of 1 → 0. A reserved suitable area is indicated by a value of 1 → 1 (Zhao et al., 2021a).

### 2.5 Division of potentially suitable areas for *P. lactiflora*

We imported the ASC file generated by Maxent into ArcGIS 10.4.1, subsequently converted it into grid data, and visualized it by overlaying it onto a map depicting China’s administrative regions. Using this foundation, we utilized the Gis reclassification function to classify the suitability of different areas for *P. lactiflora.* The corresponding area calculations were then conducted using the grid computation tool. To ascertain P. lactiflora’s habitat suitability index, we apopted the natural breakpoint classification method. The suitability of habitats was categorized into four categories: unsuitable areas (0–0.49), low suitability areas (0.49–0.55), moderately suitable areas (0.55–0.65), and highy suitable areas (0.65–1).

### 2.6 Migration of the centroid

The SDM toolbox, a Geographic Information System (GIS) application developed on a Python platform, was used to calculate the shifting patterns in the relevant areas. The centroids of future and existing suitable zones were then compared (Brown, 2014). We utilized the SDMTool package to calculate the position of the center of gravity in the suitable area for *P. lactiflora* under both current and future climate scenarios, and utilized the geosphere package (https://cran.r-project.org/package=geosphere) to quantify the range shift distance of the centroids across different climate scenarios. The distribution data layers representing both present and future conditions were superimposed using the ArcGIS overlay tool. To establish the designated set, reclassify the new layers, and categorize the suitability classes to produce the final peony suitability zoning map, this tool merges several raster data into a single output. The two suitability classes—general suitability area and most appropriate area—were considered in the calculation of the overall suitability area in this study. To examine the connections between environmental variables and the possible distribution area, the values of the predominant environmental variables and suitability degrees in different time periods were taken from 291 distribution points (Zhao et al., 2021b; Wang et al., 2023a).

### 2.7 Marxan model construction

Marxan is a multi-objective system protection planning software based on simulated annealing that is capable of achieving the most effective protection objectives within specified protection cost constraints. Due to the continuous refinement of its methodology, it has become widely used in recent years for the planning of terrestrial conservation systems (Zhang X. et al., 2021). In this study, we utilized the spatial optimization model Marxan to design the reserve network of *P. lactiflora*. We selected a grid of 25 km × 25 km as the spatial planning unit to cover China. Furthermore, in ArcGIS, we created a species distribution matrix using zone statistics tools to determine the distribution region of the target species within each planning unit (Wang et al., 2023b). Following the 10%-30% threshold outlined in the IUCN Terrestrial Ecosystem Conservation Plan (TECP), we chose 30% of the upper limit of the species’ distribution range as the optimal conservation ratio (Guo et al., 2018). The clustering of planning units within a conservation priority area is controlled by a parameter known as the boundary length modifier (BLM). The appropriate BLM value balances the boundary length of a conservation priority area with the overall cost of protecting the planning units (McDonnell et al., 2002). In this study, we selected a BLM value of 10000, an SPF value of 100, and ran the model for 100 iterations to obtain the optimal solution for the spatial planning unit. Subsequently, we imported the results into ArcGIS to construct a spatial planning map of the *P. lactiflora* reserve.

## 3 Results and analysis

### 3.1 Model optimization

**Figure 2:**
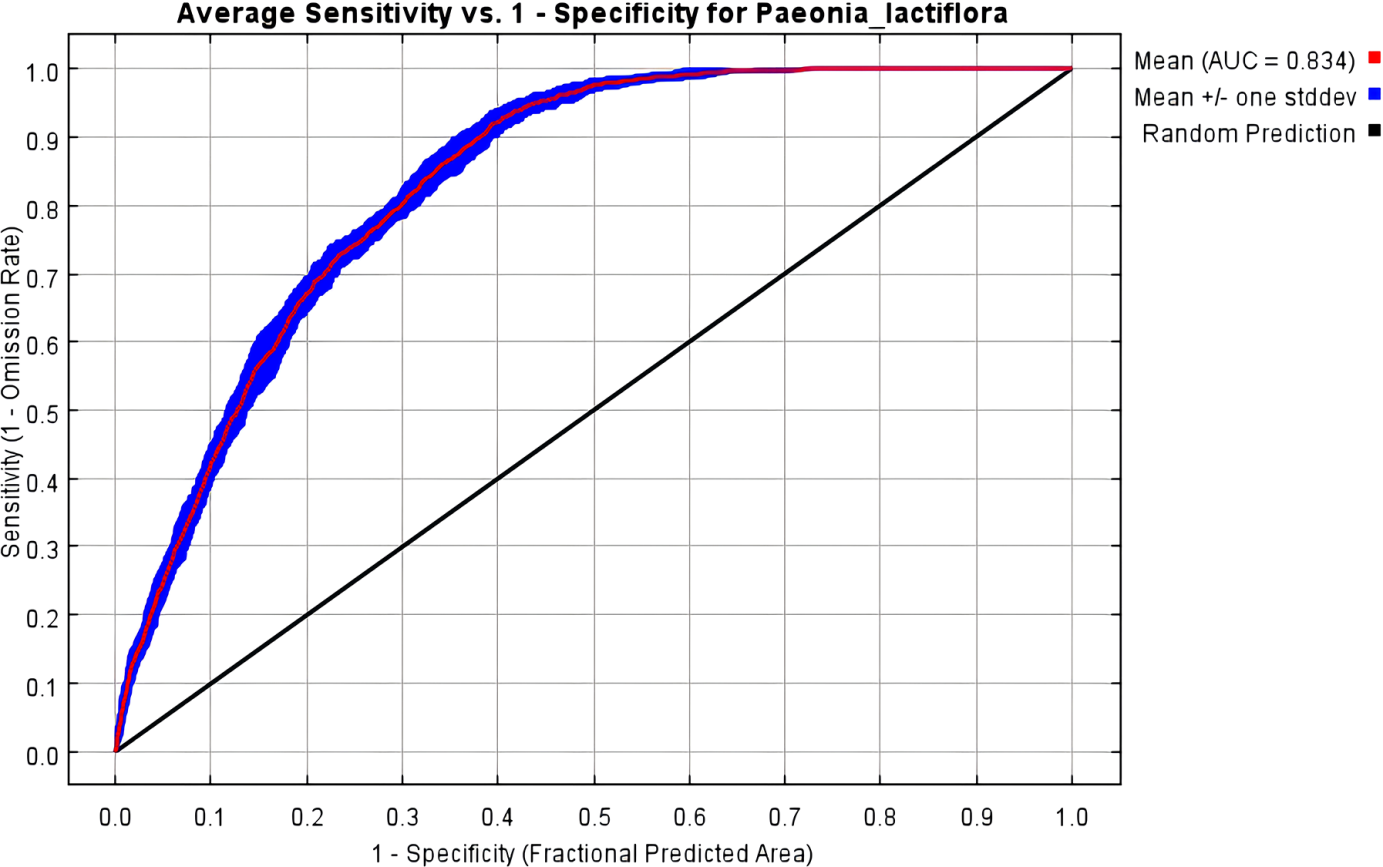
ROC response curve under the Maxent model.

The Maxent model may lead to overfitting when predicting the potential distribution of a species. We utilized the ENMeval package to cross-validate the tuning of the model using 291 distribution locations of *P. lactiflora* and 12 environmental factors across various combinations of RM and FC. When FC = LQHPT, delta, and RM = 1 are the default parameters for the model. When RM = 2.5, FC = H, and delta is considered, the AICc value is 43.81. The model is deemed optimal when AICc = 0, based on AIC criteria. Compared to the typical settings, changes were observed in the average difference (avg.diff) values. The optimized model’s area under the curve (AUC) and average test odds ratio for the top 10 percent of predictions (avg.test.or10pct) decreased by approximately 46.8% and 51.7%, respectively (**Table 2**). The optimized models exhibited decreased complexity and overfitting, leading to enhanced model fitting. Consequently, RM = 2.5 and FC = H were chosen as the final parameter settings for this study. The Maxent model was run using these optimal model parameter settings and cross-validated 10 times, resulting in an average AUC of 0.834 (**Figure 2**) and a TSS value of 0.71, which indicate accurate predictions.

**Table 2:** Maxent model’s evaluation outcomes for various parameter setting.

Model evaluation	Feature combination	Regularization multiplier	Value of delta Akaike information criterion corrected	auc.diff.avg	10% Training omission rate
Default	LQHPT	1	43.81	0.111	0.294
Optimized	H	2.5	0	0.059	0.142

### 3.2 China’s current *P. lactiflora* potentially suitable regions

**Figure 3:**
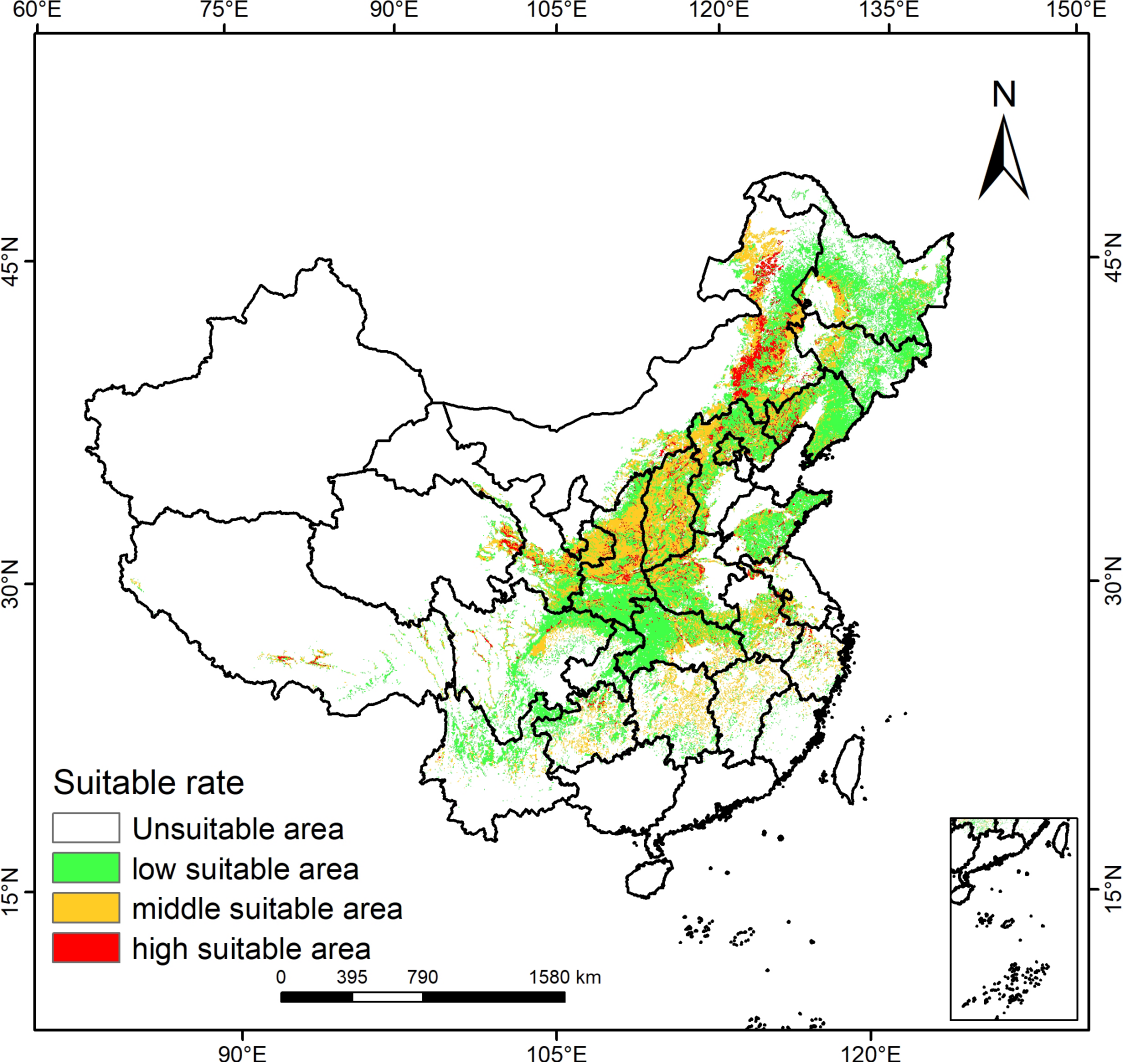
Prediction of the potentially suitable area for *P. lactiflora*.

In light of the optimized Maxent model’s forecast outcomes, the distribution map of *P. lactiflora* was divided using ArcGIS to visualize the potentially suitable distribution area across different time period, as shown in **Figure 3**. On the map, the color coding indicates the suitability levels for *P. lactiflora*, with red denoting highly suitable area, yellow denoting moderately suitable area, and green denoting low suitable habitat area, spanning 159,331 km^2^, 804,093 km^2^, and 1,347,579 km^2^, respectively. The total suitable area accounts for approximately 24.1% of the national territory and is predominantly distributed across 24 provinces and municipalities, such as Heilongjiang, Jilin, Liaoning, Inner Mongolia, Hebei, Shanxi, Shaanxi, Gansu, Qinghai, Shandong, Henan, Hubei, Anhui and Sichuan. Northeastern Inner Mongolia, eastern Qinghai, southeastern Gansu, and central Shaanxi constitute the primary regions where the highly suitable areas are located, representing 1.66% of the nation’s total area. Shanxi, Ningxia, and central Shaanxi are the main areas where the moderately suitable habitats are found, accounting for 8.38% of the nation’s total area.

### 3.3 Prediction of suitable areas for *P. lactiflora* under future climatic conditions

**Figure 4:**
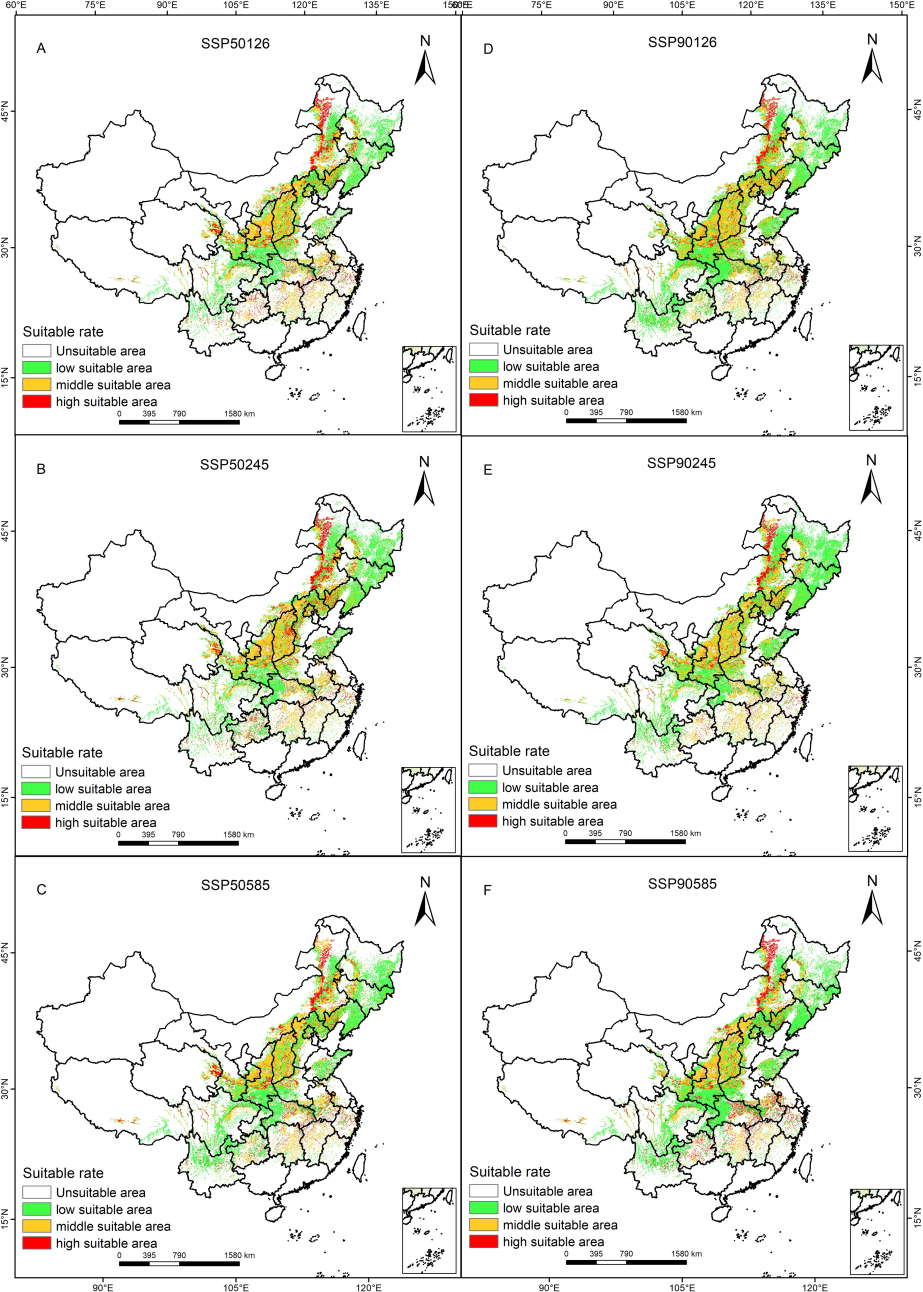
Future climatic scenarios and *P. lactiflora*’s potential distribution.

Based on the same criteria as previously mentioned, the optimized Maxent model was used to predict the potentially suitable distribution areas of *P. lactiflora* in the 2050s and 2090s, taking into account the SSP126, SSP370 and SSP580 scenarios. In addition to the area and variations within each acceptable range, we also obtained a spatial distribution map (**Figure 4**) of the projected future potentially suitable areas of *P. lactiflora* (**Table 3**).

**Table 3:** Based on the current period, the change shift in the suitable habitat of *P. lactiflora* under various climatic scenarios.

	Current (×10^4^ km^2)^	2050s (%)			2090s (%)		
		SSP126	SSP245	SSP585	SSP126	SSP245	SSP585
Unsuitable area	728.90	5.13	3.76	3.60	0.96	1.68	3.08
Low suitable	134.76	-27.5	-24.25	-18.15	-10.51	-11.90	-11.96
Moderately suitable	80.41	-12.05	-6.63	-11.59	3.42	-0.11	-21.83
Highly suitable	15.93	59.20	66.54	47.21	27.56	24.23	70.56
Total suitable	231.10	-16.17	-11.86	-11.36	-3.04	-5.31	-9.71

The bluer the color, the greater the reduction. The darker the red, the greater the increase.

When comparing the 2050s and 2090s to the current period, the area of each suitability class decreases to varying degrees for each of the three climate scenarios. The SSP126, SSP245, and SSP585 scenarios, for instance, saw their areas shrink by 16.17%, 11.86%, and 11.36%, respectively, in the 2050s. However, it’s noteworthy that the suitable area gradually increases with higher radiation forcing within this timeframe. Conversely, in the 2090s, the suitable area decreased as radiation forcing intensified. There is a notable increase in both highly suitable areas and unsuitable areas, accompanied by a decrease in low suitable area. Under the SSP585 scenario, in the 2090s, the highly suitable area expands by up to 70.6%. Over time, the overall surface area of potentially suitable distribution for *P. lactiflora* tends to decrease initially, followed by an increase, but it never surpasses the suitable area observed in the current period.

### 3.4 Dominant environmental factors

A jackknife test was used to ascertain the mean contribution of each parameter with the aim of identifying important environmental variables. The results indicated that precipitation during the wettest month was the foremost environmental factor influencing the distribution of *P. lactiflora*, contributing 43.2% (**Table 4**). Other notable factors encompassed precipitation in the warmest quarter (15.7%), slope variability (6%), maximum temperature in the warmest month (6.3%), minimum temperature in the coldest month (7.2%), and exchangeable sodium salt (9.9%). Collectively, these six environmental factors constituted 88.3% of the overall contribution, with precipitation in the wettest month (43.2%) being the most predominant. The factors exhibiting over 10% importance were precipitation in the warmest quarter (29.3%), precipitation in the wettest month (21.9%), slope variability (11.5%), maximum temperature in the warmest month (10.5%), and minimum temperature in the coldest month (10.5%). From the important values and contributions, it is evident that the factors affecting precipitation, temperature, and slope variability exerted the greatest effects. In contrast, soil characteristics and elevation had minimal influence on the distribution pattern *P. lactiflora*.

**Table 4:** Contribution rates and importance values of environmental factors.

Environmental factor	Contribution(%)	Importance (%)
Max Temperature in Warmest Month	6.3	10.5
Min Temperature in Coldest Month	7.2	10.5
Precipitation in Wettest Month	43.2	21.9
Precipitation in Driest Month	0.4	0.5
precipitation in warmest quarter	15.7	29.3
precipitation in coldest quarter	0.1	1.4
Altitude	1.9	0.7
Slope variability	6	11.5
Basic saturation	2.7	0.6
Cation exchange capacity of cohesive soil	2.5	2.2
Exchangeable sodium salt	9.9	4.5
Silt content in topsoil	4	6.2

**Figure 5:**
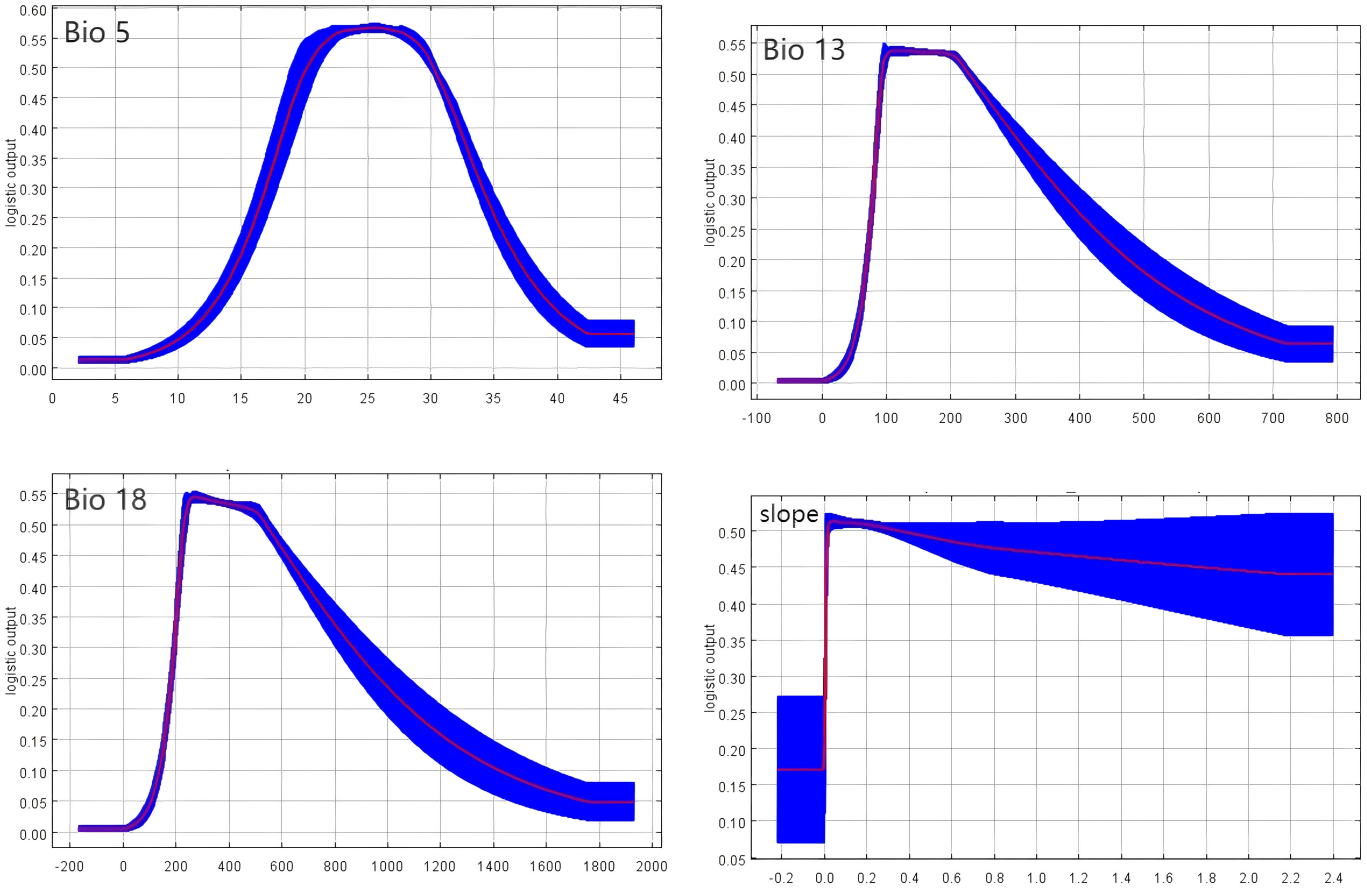
Response curve of the current climate.

Further analysis of response curves for five environmental factors that significantly impact *P. lactiflora*’s global distribution provided insights into the climatic characteristics of potentially suitable areas, considering current climatic conditions. Generally, environmental conditions are deemed favorable for plant growth when the survival probability exceeds 0.5. Our findings indicate striking similarities between the precipitation curves for the wettest month (bio13) and the warmest quarter (bio18). Both curves initiate at 0, rise with precipitation, and reach their peaks at 100 mm and 240 mm, respectively. Following the attainment of these peaks, the survival rate stabilizes within a specific precipitation range. However, beyond a certain precipitation threshold, the survival rate of *P. lactiflora* experiences a rapid decline. The slope variability curve steeply increases to attain a maximum at 0, and as it continues to ascend, the survival rate of *P. lactiflora* decreases, albeit in a relatively gradual manner. Similarly, the maximum temperature in the warmest month (bio5) possesses an ideal range, contributing to the overall survival of the species. Consequently, the thresholds for the primary environmental parameters in the projected potentially suitable area of *P. lactiflora*, as shown in **Figure 5**, are as follows: precipitation in the wettest month ranging from 10 mm to 300 mm, the precipitation in the warmest quarter ranging from 220 mm to 530 mm, maximum temperature in the warmest month ranging from 20°C to 30°C, and slope variability ranging from 0 to 0.4×10^6^.

### 3.5 Dynamic shifts in *P. lactiflora*’s appropriate habitats under various climatic scenarios

**Figure 6:**
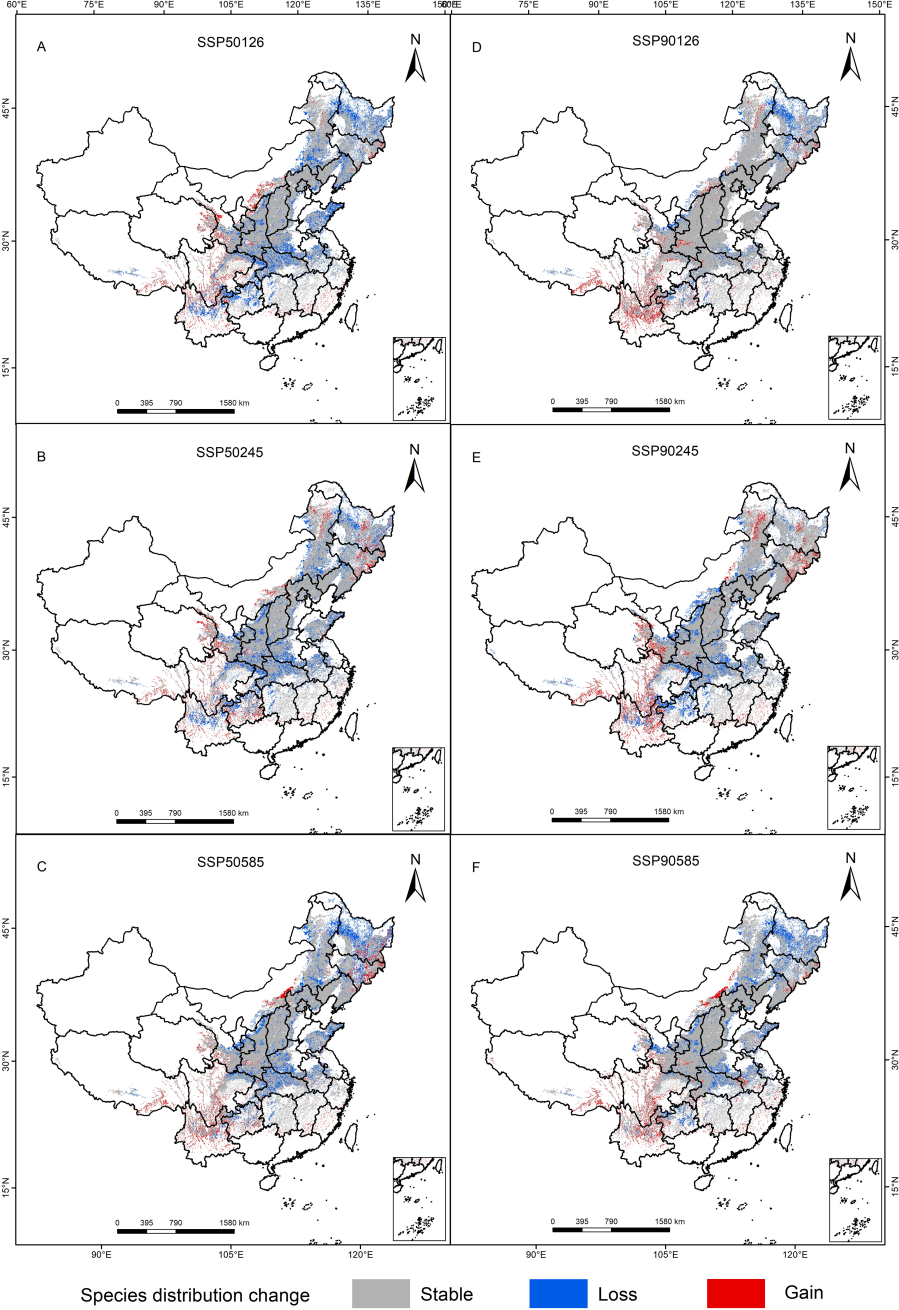
Dynamic change map of the predicted potentially suitable area of *P. lactiflora*.

The spatial patterns of *P. lactiflora* habitats under six different future climate scenarios were investigated in comparison with those of current suitable areas. The results showed that *P. lactiflora* will experience differential reductions in suitable areas during future periods and under various climate scenarios (**Table 5**). Nevertheless, the vast majority of the current suitable area will still remain suitable. The percentage of *P. lactiflora* suitable habitat lost in each of the three future climate scenarios varies from 10.47 to 22.08%, and the area of habitat loss ranges from 24.19-51.02×10^4^ km^2^. Habitat loss is mainly concentrated in northeastern China, northern and central China, including Heilongjiang, Henan, Hubei and other provinces (**Figure 6**). The increase in the extent of the region suitable for *P. lactiflora* increased by 13.66-21.67×10^4^ km^2^, with a growth rate of 5.91-9.38%. The expansion of suitable areas was primarily observed in the southwestern region, including Tibet, Sichuan, Qinghai, and Yunnan provinces. The regional rates of change ranged from 3.04% to 16.17%, with the lowest rates observed under the SSP126-2090s climate scenario and the highest rates under the SSP126-2050s climate scenario. Specifically, the SSP126-2050s climate scenario also showed a maximum loss of 51.02 × 10^4^ km^2^ of suitable area and a minimum gain of 13.66 × 10^4^ km^2^, indicating a more sensitive response to climate change under this scenario. However, the loss of area under the SSP126-2090s climate scenario was relatively minimal. Our comparative analysis of the changes in spatial patterns of potential habitat for *P. lactiflora* under various future climate change scenarios, the rates of change in spatial regions were higher in the 2050s than in the 2090s, indicating that the pattern of spatial changes in habitat was more significant in the 2050s.

**Table 5:** *P. lactiflora* changing distribution area under various time periods and scenarios.

Period	Climate scenario	Habitat area (×10^4^km^2^)	Loss (×10^4^km^2^)	Stable (×10^4^km^2^)	Gain (×10^4^km^2^)	Species range change(%)	Percentage loss (%)	Percentage Gain (%)
Current		231.10						
	SSP126	193.74	51.02	180.08	13.66	-16.17	22.08	5.91
2041-2060	SSP245	203.69	44.54	186.56	17.13	-11.86	19.27	7.41
	SSP585	204.84	44.64	186.46	18.38	-11.36	19.32	7.95
	SSP126	224.06	24.19	206.91	17.15	-3.04	10.47	7.42
2081-2100	SSP245	218.83	33.94	197.16	21.67	-5.31	14.69	9.38
	SSP585	208.67	38.71	192.39	16.28	-9.71	16.75	7.04

### 3.6 Centroid changes of *P. lactiflora* based on future climatic scenarios

**Figure 7:**
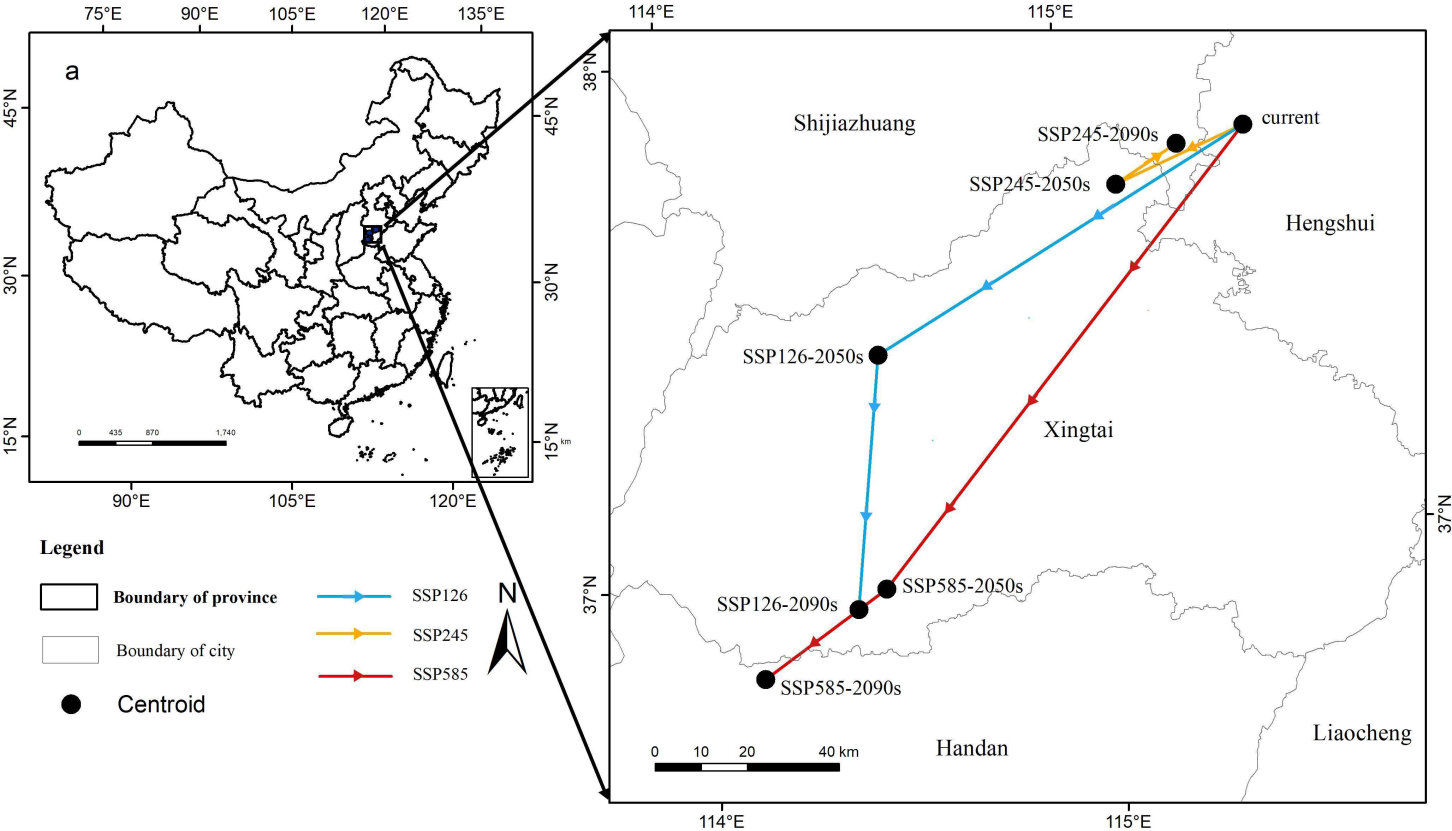
Centroids of the predicted potentially suitable area of *P. lactiflora*.

We used a centroid to delineate the central point of the distribution area, serving as a proxy for the location of the *P. lactiflora* habitat (**Figure 7**). The results indicated that, despite some variations in the shifts of suitable areas across different climate scenarios, the primary migration trend remained relatively consistent, predominantly towards the southwest. All of the aforementioned areas were situated within Hebei Province. Presently, the potential geometric centroid of the *P. lactiflora* habitat is situated around Taocheng District, Hengshui City, Hebei Province, China (115.46°E, 37.78°N). Under the SSP585 climate scenario in the 2090s, the centroid of the suitable growing region for *P. lactiflora* shifted to its southernmost boundary. At this point, the migration distance amounted to 159,071 meters, positioning the centroid of the suitable growing area in Handan, Hebei Province (114.14°E, 36.81°N). Under both the SSP126 and SSP585 climate scenarios, it was predicted that the migration distance of centroid within the potentially suitable area would increase with escalating radiative forcing. Conversely, under the SSP245-2050s climate conditions, the centroid of suitable area shifted southwest. During this period, the centroid was situated in NingJin County, Xingtai City, Hebei Province (115.12°E, 37.69°N), with a migration distance of 309,85 meters. However, in the suitable area for *P. lactiflora* under the SSP245-2090s climate scenario, the centroid shifted northeast. The new location, with a migration distance of 15,331 meters, was in Shijiazhuang City, Hebei Province (115.28°E, 37.76°N).

### 3.7 Priority protected regions of *P. lactiflora* under current climate scenarios

**Figure 8:**
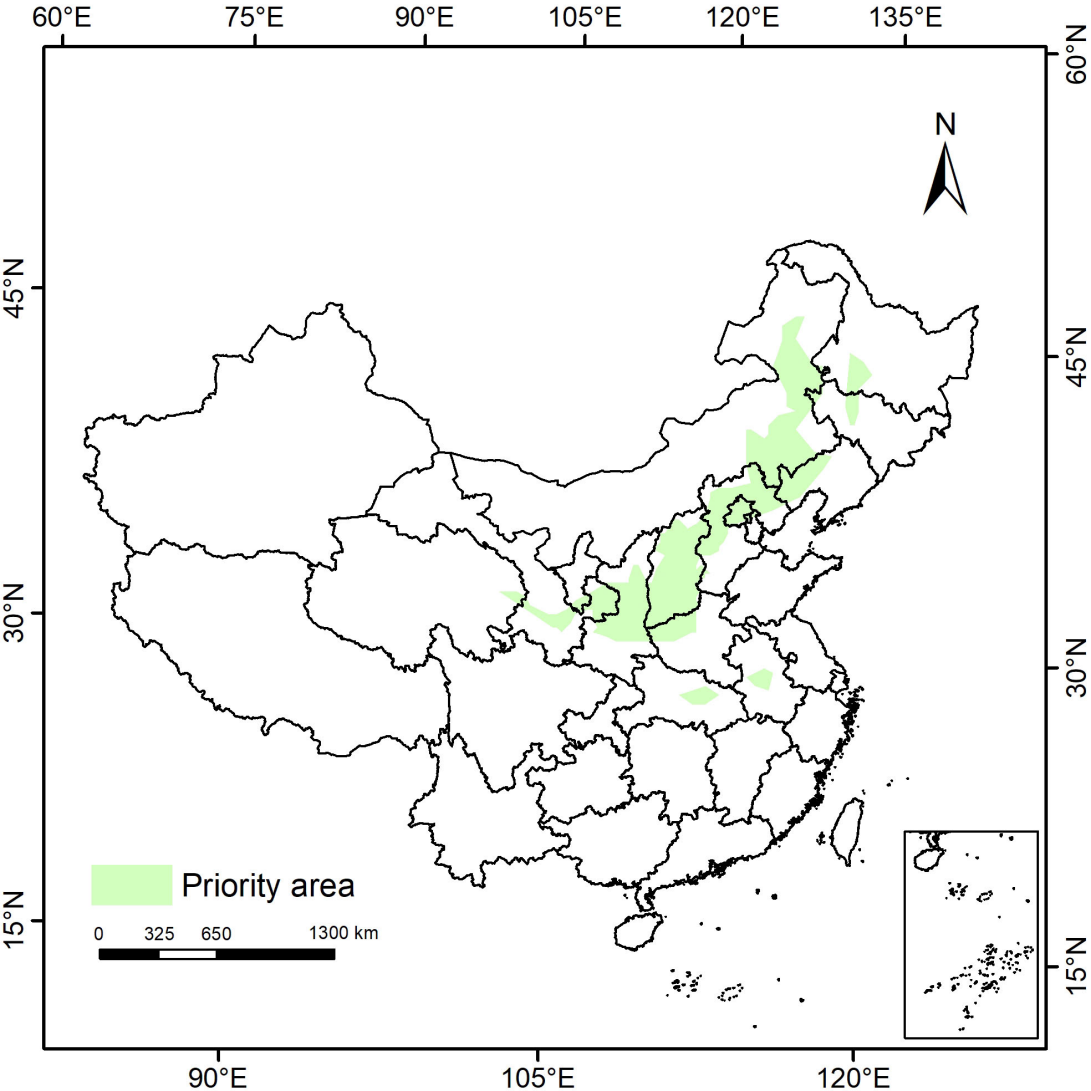
Priority protected areas of *P. lactiflora* in China predicted by the Marxan model.

We conducted an investigation into the potential distribution range of *P. lactiflora* in the contemporary climatic conditions. We utilized the Marxan model to identify the priority conservation areas and subsequently imported the computational results from ArcGIS software to formulate a comprehensive conservation strategy with *P. lactiflora* as the central focus (**Figure 8**). The findings revealed that protected areas (PAs) were predominantly situated in 13 provinces, spanning across eastern Inner Mongolia, the border regions of Heilongjiang and Jilin, northern Liaoning, northern Hebei, Shanxi, central Shaanxi, southern Gansu, eastern Qinghai, central Hubei, and central Anhui. These areas substantially overlapped with the potential altitude zones and moderately adapted habitats for *P. lactiflora* under current climatic conditions, thereby enhancing the credibility of the predictive results.

## 4 Discussion

### 4.1 Evaluation of Maxent after optimization

The Maxent model is independent of the sample size and can generate species response curves to quantify environmental factors in suitable habitats (Phillips et al., 2006; Gebrewahid et al., 2020). Many researchers have demonstrated that the default settings of Maxent can be intricate, leading to a high degree of overfitting. This may reduce the accuracy of the results and make them more difficult to interpret, particularly for response curves representing highly fluctuating environmental conditions. In this study, based on three environmental variables—climate, topography, and soil factors—the ENMeval package in R was used to optimize the model to mitigate overfitting and sampling bias, thereby improving prediction accuracy (Sony et al., 2018). This approach restricts the background data to the region corresponding to the calibrated location to ensure that the potential geographic distribution area encompasses the current distribution points. By adopting this strategy, the performance of the Maxent model can be enhanced by adjusting its parameters. The accuracy of the system can be assessed by improving the alignment between the projected and actual distribution areas and visually inspecting the Geogrid map (Guo et al., 2018). To minimize errors, we evaluated 48 parameter combinations in the ENMeval package by constructing eight numerical regularization multipliers with values ranging from 0.5 to 4 and using six feature combinations. The results indicated that the optimal Maxent model settings were FC = H and RM = 2.5. Model complexity affects the transferability of species distributions. Studies have shown that the complexity of the Maxent model can be controlled by utilizing the AICc parameter and adjusting the regularization multiplier (Li et al., 2020; Warren and Seifert, 2011). When the model was optimized, the AICc decreased from 43.81 to 0, indicating reduced overfitting after optimization. The parameter-optimized Maxent model effectively predicted the distribution of *P. lactiflora*. In accordance with Shelford’s tolerance law, the response curve was noticeably smoother and more akin to a normal distribution curve (Zhao et al., 2021a). The optimized Maxent model simulated the potential distribution regions of *P. lactiflora* with high reliability.

The area under the ROC curve (AUC) is a crucial evaluation metric, particularly for binary classification problems. The AUC measures a model’s ability to rank samples, specifically, its capacity to prioritize positive samples over negative ones. This is particularly important for species distribution predictions using the Maxent model. A Maxent model with a high AUC value indicates its ability to predict the presence or absence of a species more accurately, thereby enhancing its practical utility in real-world applications. Furthermore, the AUC value is independent of the decision thresholds and can be used to compare different models, making it the current optimal metric for assessing model prediction accuracy (Swets, 1988; Chen et al., 2012). In this study, the AUC value exceeded 0.8, indicating that the modeling of the current potential distribution area provides a reasonable basis for predicting the future potential distribution of *P. lactiflora*. However, it should be noted that the AUC value largely depends on the sample size and the coverage of the climatic niche within the relevant region. Dyderski et al. (2018) suggested that, after resampling, the AUC value tends to decrease as the sample size increases. As the proportion of climatic variation occupied by the species increases, the distinction between occupied and unoccupied areas diminishes. Conversely, models assessing rare species with few records typically exhibit higher AUC values because their narrow climatic niche often contrasts sharply with that of the surrounding areas. Chen et al. (2012) demonstrated that when the sample size was relatively small, the AUC values of species distribution models fluctuated more and exhibited poorer stability. When the sample size exceeded 120, the AUC value became increasingly stable and eventually remained largely unchanged, even with variations in the sample size.

### 4.2 Effects of environmental variables on geographical range of *P. lactiflora*

It is crucial to understand the major environmental factors shaping the spatial distribution patterns of species from an ecological perspective. The distribution of suitable areas for a species is primarily influenced by several factors, including precipitation in the wettest month, precipitation in the warmest quarter, exchangeable sodium salt, maximum temperature in the warmest month, minimum temperature in the coldest month, and slope variability. These conclusions were supported by the percentage contribution, permutation importance, and jackknife test results from the Maxent model. In this study, the high-suitability areas were mainly concentrated in northeastern Inner Mongolia and other regions, which aligned with the optimal growth zone of *P. lactiflora.* The accuracy of these results has been verified (Li et al., 2019).

The geographical distribution of plants is greatly influenced by hydrothermal conditions, and terrestrial plants are particularly sensitive to temperature and precipitation (Bradie and Leung, 2017). The findings of the Maxent model indicate that temperature and precipitation were the two most crucial environmental variables limiting the potential distribution of *P. lactiflora*, contributing a combined total of 72.9% of the model predictions. Previous studies have indicated that temperature plays a considerable role in the morphological structure, vegetative growth, seed dormancy, and germination of *P. lactiflora*. The optimal temperature range for the growth of *P. lactiflora* is 15–25°C. When *P. lactiflora* plants are subjected to high-temperature stress, there is a reduction in the rate of chlorophyll synthesis and an acceleration of chlorophyll degradation within the plants. Simultaneously, this stress results in the accumulation of reactive oxygen species in plants. Furthermore, when exposed to high-temperature stress, the leaves of *P. lactiflora* exhibit a yellow-green coloration, accompanied by small and dense sunburn-induced perforations and large dark brown patches. These effects ultimately cause plant wilting and death. As a typical temperate plant, *P. lactiflora* thrives under temperate climatic conditions but is intolerant of high humidity or high temperatures. The average temperature of the coldest month in its distribution areas generally ranges from -5°C to -20°C, or even lower (Shen et al., 2012; Hou et al., 2023). Precipitation regulates plant carbon sequestration and transpiration by influencing soil moisture (Shi et al., 2023). When the precipitation in the wettest month fell below 90 mm, or the precipitation in the warmest quarter was less than 220 mm, the probability of the presence of *P. lactiflora* significantly decreased. This indicates that precipitation is a crucial limiting factor for its distribution. Previous studies have shown that as water availability decreases, photosynthesis by *P. lactiflora* notably diminishes, accompanied by external morphological changes such as yellowing and defoliation (Wang et al., 2014). The emergence and growth of *P. lactiflora* are directly influenced by water availability (Zhang et al., 2018). These hydrological factors are likely to play a primary role in shaping the ecological adaptations of *P. lactiflora* and affect numerous physiological processes related to seed germination, growth, and plant development. Consequently, they have a substantial impact on species distribution. Zhang et al. (2022) showed that the amount of active components in *P. lactiflora* roots was significantly influenced by annual precipitation and annual average temperature. In the present study, the survival rate of *P. lactiflora* was higher in regions with lower precipitation and colder temperatures (Shen et al., 2012).

Topography and soil were also important factors influencing the distribution of *P. lactiflora*, with a total contribution of 27.1%. Lv et al. (2009) reported that wild peony species are primarily distributed in mountainous grasslands and under forest canopies at elevations ranging from 480 to 700 m on hillsides in Northeast China. They are also found at specific elevations in hilly, mountainous, and plateau regions, such as North China, the Qinling Mountains, the Qilian Mountains, and the Yinshan Mountains. Additionally, these species are present in mountainous grasslands at elevations of 1000–2300 m in other Chinese provinces. Relevant studies have shown that factors such as slope gradient and aspect not only influence plant distribution and community structure but also affect the distribution of plant biomass and root system architecture (Ferrari et al., 2021). This suggests that geographic factors, such as elevation, topography, slope, and slope direction, have a significant influence on the distribution of *P. lactiflora*. Inorganic elements in the soil such as Mn, Fe, Zn, and Cu play crucial roles in the growth of *P. lactiflora*. Among these, Zn plays a key role in the formation of *P. lactiflora* (Chen et al., 2009). Therefore, temperature, precipitation, topography, and soil factors influence the potential distribution pattern of Paeoniaceae. However, in this study, precipitation had a greater influence than temperature and soil topography among the environmental factors affecting its distribution.

### 4.3 Changes in the potential geographical distribution and resource protection of *P. lactiflora*

It is crucial to quantify the impact of climate change on the potential distribution patterns of species to create conservation plans that preserve ecological balance (Deb et al., 2017). The response of *P. lactiflora* distribution to climate change was primarily evident in the varying degrees of shrinkage of suitable areas under future climate scenarios. This included the expansion of highly suitable areas, a reduction in less suitable areas, and significant intensification of habitat fragmentation. Moderately suitable areas decreased under all scenarios, except for an increase under the SSP585-2090s climate scenario. Under future climate scenarios, the distribution of *P. lactiflora* will not only decrease in terms of suitable areas but also in its spatial patterns. The boundaries of suitable areas will change drastically, and the rate of loss of suitable areas for *P. lactiflora* will be significantly higher than the rate of increase of new suitable areas. These results suggest that suitable areas for *P. lactiflora* will be found at lower latitudes and higher elevations in the future. This trend aligns with the projected southwestward migration of the center of mass under future climate scenarios.

Wild populations of *P. lactiflora* are now endangered due to the destruction of their natural habitats and overexploitation of the environment. Considering projected future climate change, and to meet the market demand for *P. lactiflora*, we recommend prioritizing the conservation of *P. lactiflora* in its primary distribution areas, such as North China and Northeast China. Given the predicted decrease in its distribution range under future climate conditions, conservation methods should involve adaptive management techniques to effectively address the effects of climate change. Furthermore, by considering various climate change scenarios and their impacts on species distribution, we can gain more nuanced perspectives on the long-term conservation prospects of *P. lactiflora*. The reliability of predictions can be enhanced by utilizing sophisticated modeling approaches and broader spatial coverage of distribution data. In summary, our research contributes to the overall understanding of *P. lactiflora* distribution and provides insights for conservation and management initiatives. By addressing the known research gaps and incorporating additional factors, we can improve the scientific rigor and applicability of future studies, ultimately aiding in the preservation of this crucial species and its habitat.

### 4.4 Potential limitations and future outlooks

We must acknowledge some limitations in the current study. We focused on climate and topography, but other factors could influence a species’ potential geographic range. In addition to affecting temperature and precipitation, climate complexity affects light radiation intensity, the soil carbon cycle, and changes in the ozone layer. Biological elements, such as competition, reproduction, and human activity, as well as abiotic physical barriers, also affect plant growth (Blois et al., 2013). Therefore, in future research, we can integrate the physiological and biochemical effects of plants, interactions between organisms, ecosystem changes, and human factors to make more accurate predictions and enhance the precision of the model. In this study, climate variables and land cover conditions were employed as model inputs. However, we anticipate that the future land cover will change (Wang et al., 2023b). To create more accurate estimates, future studies should examine the complexity of climate change effects in greater detail.

Climate change indirectly influenced the population composition and distribution of *P. lactiflora* by affecting the ecosystem. In this study, only two time periods, the 2050s and the 2090s, were considered for environmental factor variables. The aim of this study was to predict the potential range of *P. lactiflora* in China. Several investigations have demonstrated that changes in research scale can lead to alterations in background data, ultimately affecting model formulation (Ji et al., 2019; Ma and Li, 2023). When adjusting the scope of the study, background data points should be carefully compared with species occurrence points, rather than relying solely on random sampling within the study region. However, identification of suitable background data points remains an area worthy of further exploration. Currently, a standardized system for assessing suitability within an appropriate area is lacking, and the choice of a threshold directly affects the delineated suitable area. This can result in substantial discrepancies between the designated highly suitable range and actual distribution. Therefore, it is crucial to select an appropriate threshold for classification and to validate it against the observed distribution. Future research could explore the overall trends in the potential geographic distribution across various time periods. Further investigation is required to incorporate as many relevant variables as feasible into computer model simulations to achieve more precise forecasts, particularly as information becomes more comprehensive and accessible in the future. Despite these limitations, our initial application of the optimized Maxent model marks the first step in macro-planning for reliably predicting potentially suitable areas in China. In future studies, we will collect data on the global and regional distribution of *P. lactiflora* to enable a more comprehensive analysis of its optimal environmental conditions. Nevertheless, the findings of this study provide a theoretical foundation for the development of practical adaptation strategies tailored to rare indigenous plant species facing climate change. These results remain highly relevant for guiding the preservation of germplasm resources, future planting initiatives, and the sustainable development and utilization of *P. lactiflora*.

## 5 Conclusions

In this study, we used an optimized Maxent model to predict, for the first time, the distribution of areas favorable for *P. lactiflora* considering current and projected climate conditions. The data revealed that *P. lactiflora* was predominantly located in Northeast and North China, covering the provinces of Inner Mongolia, Hebei, and Shanxi, totaling 231.1 × 10^4^ km^2^. Precipitation in the wettest month (100–230 mm), precipitation in the warmest month (240–540 mm), the coldest month’s minimum temperature (≤4°C), and the warmest month’s maximum temperature (20–30°C) emerged as the most critical factors influencing its distribution. Temperature was the second most significant factor. The potential habitat of *P. lactiflora* is projected to shift toward the southwest in the 21st century to allow it to adapt to global warming. However, unlike the current climate conditions, all future scenarios are expected to lead to varying degrees of reduction in suitable areas for *P. lactiflora* because of its sensitivity to moisture and temperature. A decline of 3.04–16.17% was predicted for the entire potentially suitable area. Conversely, it is expected that, under all circumstances, the area of highly suitable habitats will expand to different extents. Notably, the highly suitable areas identified by the Maxent model were aligned with the key conservation sites for *P. lactiflora*, primarily situated in North and Northeast China. This study offers valuable insights into the potential distribution of *P. lactiflora* under different climate scenarios, considering various environmental factors. The outcomes of this study provide a scientific basis for the management, conservation, and strategic site selection of *P. lactiflora*, while enhancing our understanding of the primary factors influencing population dispersal.

## Data Availability

The datasets presented in this study can be found in online repositories. The names of the repository/repositories and accession number(s) can be found in the article/**Supplementary Material**.
