# Supplementary figures and images for: Prediction of the potentially suitable areas of Paeonia lactiflora in China based on Maxent and Marxan models
Source: Front Plant Sci. 2025 Jan 9;15:1516251. doi: 10.3389/fpls.2024.1516251 (PMC11754415; doi:10.3389/fpls.2024.1516251)

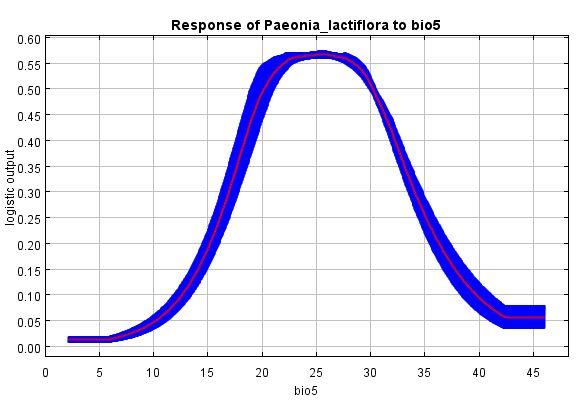

Supplement: Supplementary file 8 [file Image1.jpeg]

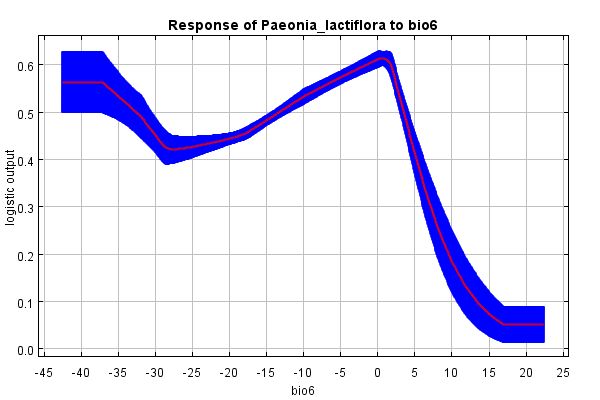

Supplement: Supplementary file 9 [file Image2.jpeg]

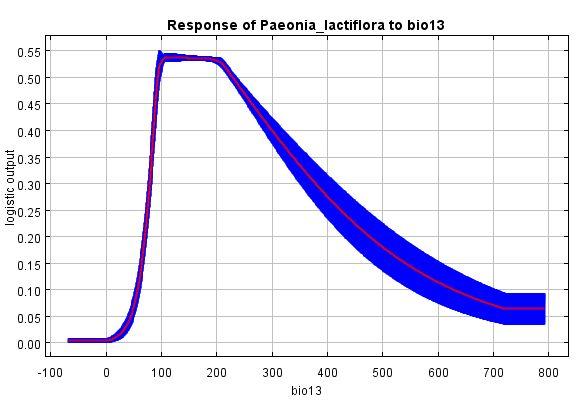

Supplement: Supplementary file 10 [file Image3.jpeg]

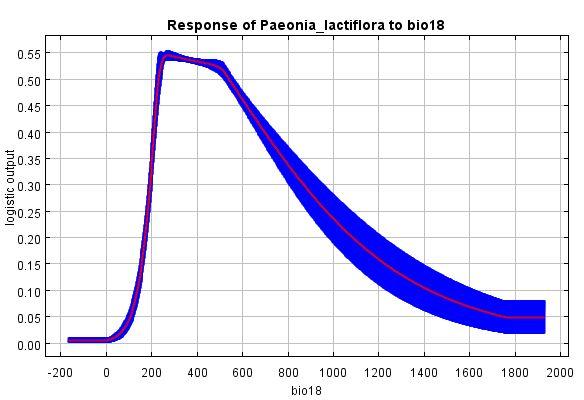

Supplement: Supplementary file 11 [file Image4.jpeg]

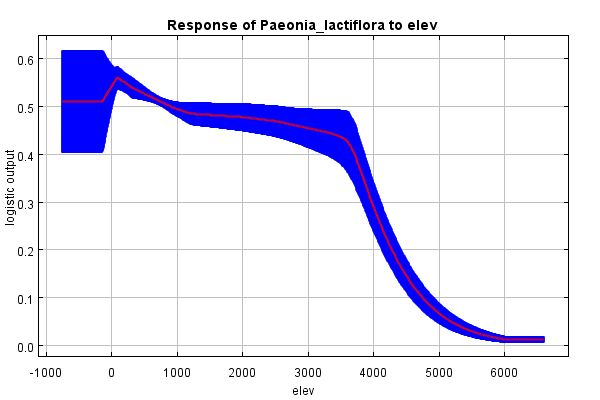

Supplement: Supplementary file 12 [file Image5.jpeg]

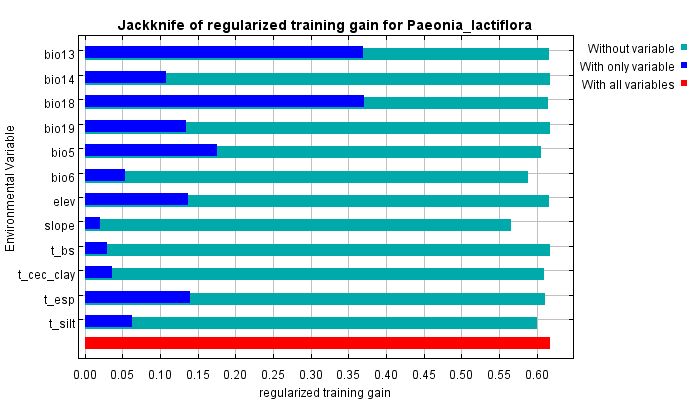

Supplement: Supplementary file 13 [file Image6.jpeg]

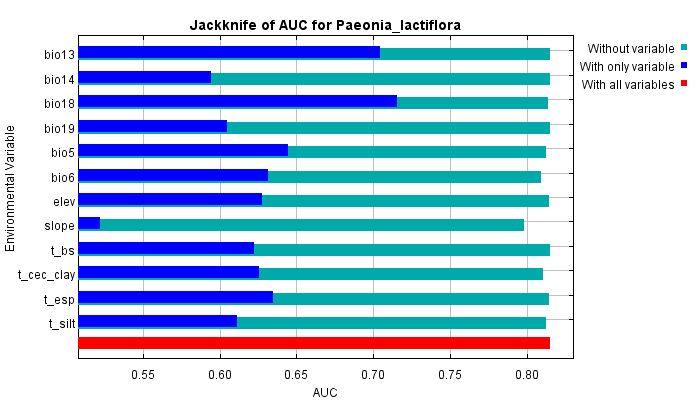

Supplement: Supplementary file 14 [file Image7.jpeg]

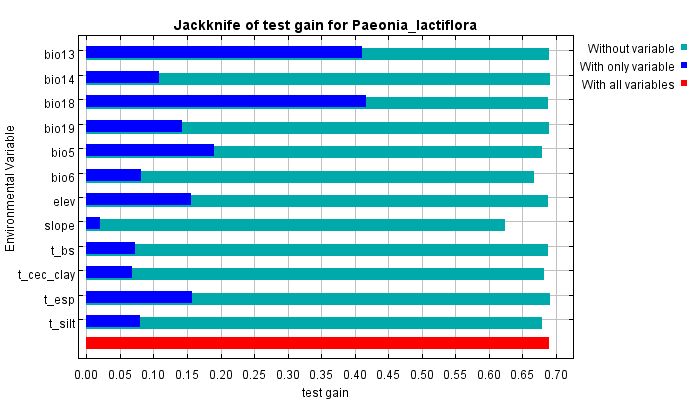

Supplement: Supplementary file 15 [file Image8.jpeg]

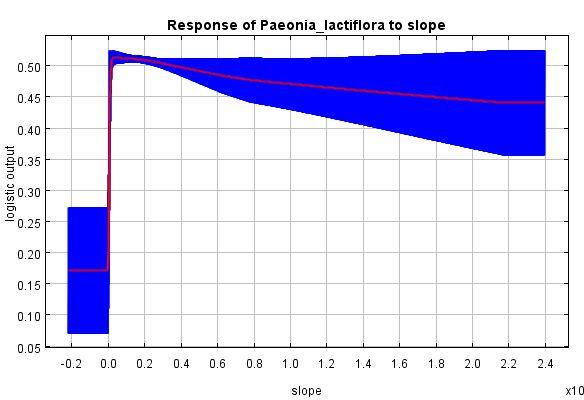

Supplement: Supplementary file 16 [file Image9.jpeg]

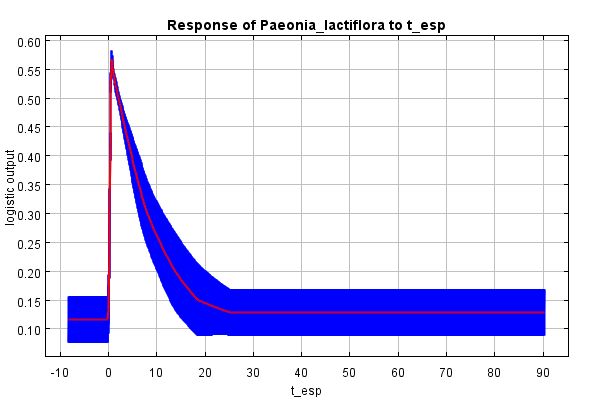

Supplement: Supplementary file 17 [file Image10.jpeg]

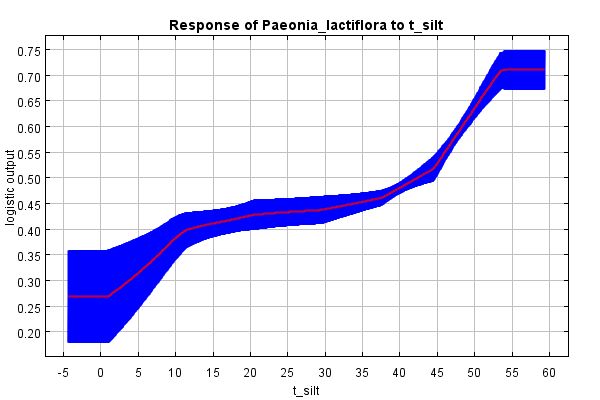

Supplement: Supplementary file 18 [file Image11.jpeg]

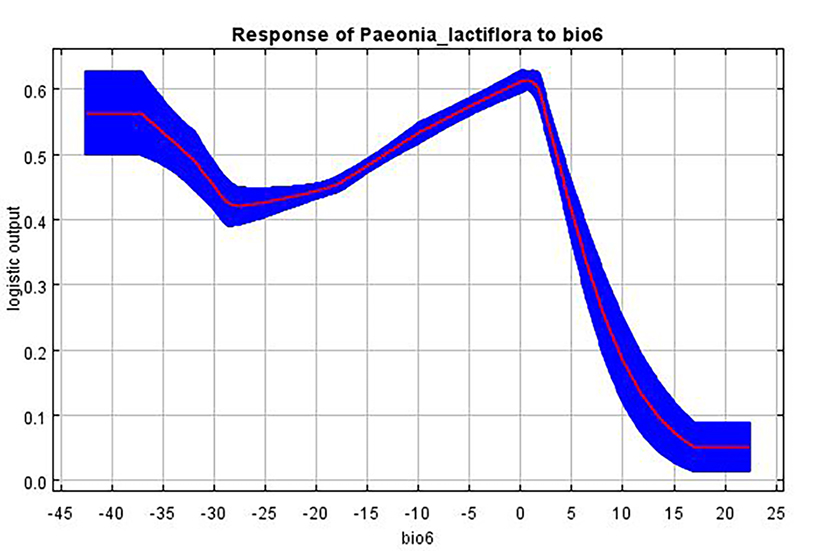

Supplement: Supplementary file 19 [file Image12.jpeg]

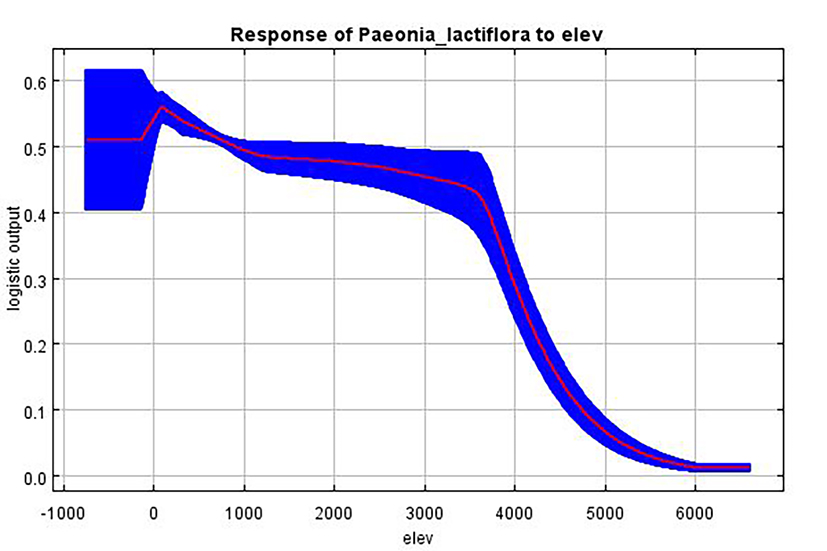

Supplement: Supplementary file 20 [file Image13.jpeg]

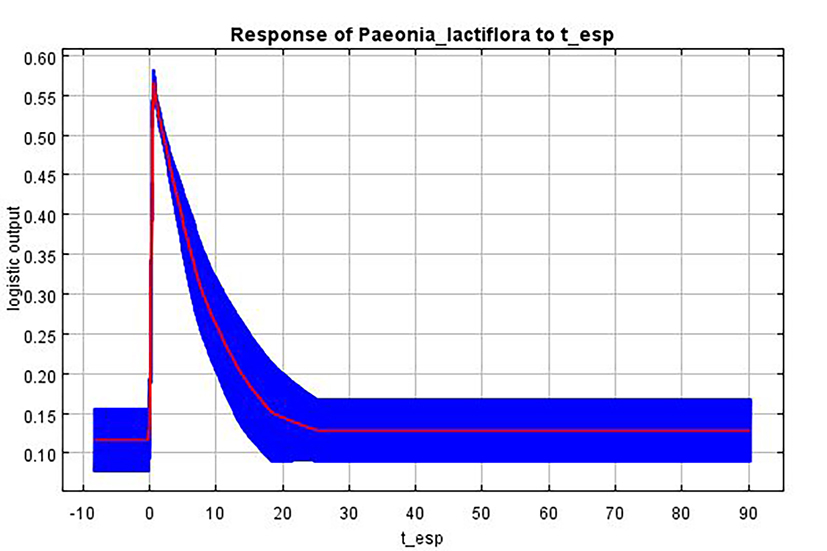

Supplement: Supplementary file 21 [file Image14.jpeg]

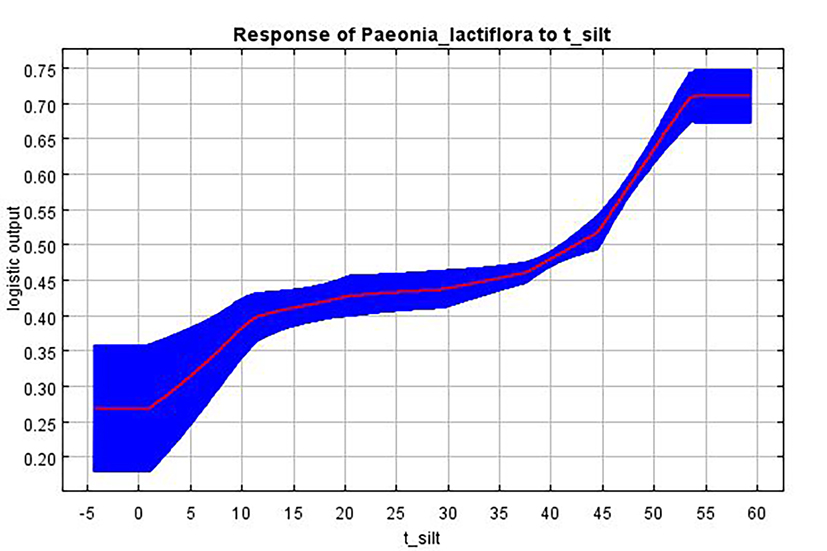

Supplement: Supplementary file 22 [file Image15.jpeg]
